# PD1/PD-L1 Expression in Blastic Plasmacytoid Dendritic Cell Neoplasm

**DOI:** 10.3390/cancers11050695

**Published:** 2019-05-19

**Authors:** Phyu P. Aung, Narittee Sukswai, Reza Nejati, Sanam Loghavi, Weina Chen, Carlos A. Torres-Cabala, C. Cameron Yin, Marina Konopleva, Xiaofeng Zheng, Jing Wang, Zhenya Tang, L. Jeffrey Medeiros, Victor G. Prieto, Naveen Pemmaraju, Joseph D. Khoury

**Affiliations:** 1 Department of Pathology, The University of Texas MD Anderson Cancer Center, Houston, TX 77030, USA; PAung@mdanderson.org (P.P.A.); reza.nejati@fccc.edu (R.N.); ctcabala@mdanderson.org (C.A.T.-C.); vprieto@mdanderson.org (V.G.P.); 2 Department of Hematopathology, The University of Texas MD Anderson Cancer Center, Houston, TX 77030, USA; NSukswai@mdanderson.org (N.S.); sloghavi@mdanderson.org (S.L.); cyin@mdanderson.org (C.C.Y.); ZTang@mdanderson.org (Z.T.); ljmedeiros@mdanderson.org (L.J.M.); 3 Department of Pathology and Laboratory Medicine, The University of Texas at Southwestern, Dallas, TX 77030, USA; Weina.Chen@UTSouthwestern.edu; 4 Department of Dermatology, The University of Texas MD Anderson Cancer Center, Houston, TX 77030, USA; 5 Department of Leukemia, The University of Texas MD Anderson Cancer Center, Houston, TX 77030, USA; mkonople@mdanderson.org (M.K.); NPemmaraju@mdanderson.org (N.P.); 6 Department of Bioinformatics and Computational Biology, The University of Texas MD Anderson Cancer Center, Houston, TX 77030, USA; XZheng2@mdanderson.org (X.Z.); jingwang@mdanderson.org (J.W.)

**Keywords:** PD-L1, immunotherapy, RNA sequencing, immune checkpoint regulation

## Abstract

Patients with blastic plasmacytoid dendritic cell neoplasm (BPDCN) have poor outcomes despite intensive chemotherapy, underscoring the need for novel therapeutic approaches. The expression status of PD1/PD-L1 in BPDCN remains unknown. We evaluated PD1/PD-L1 by immunohistochemistry and RNAseq expression profiling in a cohort of BPDCN patients. The study group included 28 patients with a median age of 66.8 years (range, 22.8–86.7), 22 men and 6 women. PD-L1 expression was detected by immunohistochemistry in 10/21 (47.6%) cases. PD-L1 expression had a median H-score of 157. The H-score was ≥60 in 7 patients. PD-L1 protein levels (H-score) were proportional to normalized PD-L1 mRNA transcript levels (CD274 mRNA). In addition, high-level PD-L1 expression correlated with higher numbers of PD1-positive cells within BPDCN tumors. There was no correlation between clinicopathologic characteristics and PD-L1 expression status. Similarly, there was no significant difference in overall survival between patients with PD-L1-positive and PD-L1-negative BPDCN (median 12 vs. 23 month, respectively; *p* = 0.743). In conclusion, PD-L1 expression by tumor cells is detectable in a sizeable subset of patients with BPDCN, suggesting that exploration of the effectiveness of therapeutic inhibition of the PD1/PD-L1 axis in patients with refractory or progressive BPDCN is warranted.

## 1. Introduction

Blastic plasmacytoid dendritic cell neoplasm (BPDCN) is an aggressive hematologic malignancy derived from plasmacytoid dendritic cells. The neoplastic cells are characteristically positive for CD4, CD56, CD123, TCF4 and TCL1 [1]. The median age of patients with BPDCN is 58 years, and men are affected more commonly than women [2]. No consensus guidelines for BPDCN therapy are available, and a variety of treatment strategies based on chemotherapy regimens designed for lymphoid and myeloid malignancies are generally pursued. In eligible patients, stem cell transplantation (allo-SCT) has been shown to result in durable remissions [3]. While novel therapeutic approaches targeting CD123 have shown promising results [4], there is a need for novel therapeutic options for patients with BPDCN.

Immune checkpoint inhibitors targeting the PD1 (programmed death 1)/PD-L1 (programmed death-ligand 1) axis have resulted in durable responses in many cancer types, often in patients with advanced-stage disease [5]. PD-L1 is an immune checkpoint regulator that plays a role in tumor evasion from host immune surveillance [6]. PD-L1 is expressed by antigen-presenting cells and indirectly induces T-cell inactivation through interaction with PD1 on T-cells [7]. In peripheral tissues, PD-L1 expression directly inactivates self-reactive lymphocytes. PD1 ligands expressed on tumor cells, including PD-L1, regulate the generation of adaptive regulatory T-cells resulting in tumor-induced immune suppression [8]. Inhibitors of the PD1/PD-L1 axis, including pembrolizumab and nivolumab, which target PD1, play a prominent role in modulating T-cell activity through the interaction with its ligands, PD-L1 and PD-L2. Immunotherapy with PD1- and PD-L1-targeted monoclonal antibodies has dramatically changed the therapeutic and prognostic landscape for several types of malignancy including treatment-refractory melanoma [9], non-small cell lung carcinoma [10,11,12,13], renal cell carcinoma [14], and Hodgkin lymphoma [15]. These responses correlate with PD-L1 expression in the tumor cells themselves [16].

Various immunohistochemistry (IHC) companion diagnostic assays have been developed for the detection of PD-L1 in formalin-fixed paraffin embedded (FFPE) tissue samples. Monoclonal anti-PD-L1 antibody clones, including 22C3 and 28-8, have been used in pivotal trials evaluating the efficacy of immune checkpoint inhibitors such as pembrolizumab and nivolumab, respectively [17]. The correlation between response to these therapies and PD1/PD-L1 expression remains incompletely understood and varies across tumor types and occasionally by disease stage [18]. In a meta-analysis of 3107 patients with various solid tumor types including lung, gastrointestinal tract, liver, and genitourinary system carcinomas, melanoma, glioblastoma, mesothelioma, and oral squamous cell carcinoma [19], it was reported that the median percentage of solid tumors with PD-L1 expression was 52.5%. Besides its utility as a biomarker, PD-L1 expression is associated with prognosis in various cancer types [6,20,21,22]. In gastric, hepatocellular, renal cell, esophageal, pancreatic, ovarian, and bladder carcinomas, PD-L1 expression is associated with worse patient outcomes. In contrast, PD-L1 expression correlates with better clinical outcomes in patients with breast and Merkel cell carcinoma. The prognostic value of PD-L1 expression in lung and colorectal cancers and melanoma remains controversial. Notably, there is no consensus on a single modality to assess PD-L1 expression, and large-scale studies of PD-L1 have used various antibody clones, scoring systems, and cutoff values.

PD-L1 expression has been shown to be constitutively expressed in dendritic cells (DC) in patients with multiple myeloma [23,24]. However, the frequency of PD1/PD-L1 expression in BPDCN, as a potential prerequisite for determining the potential utility of PD1/PD-L1 blockade in this disease, remains unknown. In addition, the prognostic significance of PD-L1 expression in BPDCN has not been reported. In this study, we surveyed PD1/PD-L1 expression in primary BPDCN tumors and correlated their expression with clinical and laboratory findings.

## 2. Results

### 2.1. PD-L1 and PD1 Expression by Immunohistochemistry

**Figure 1:**
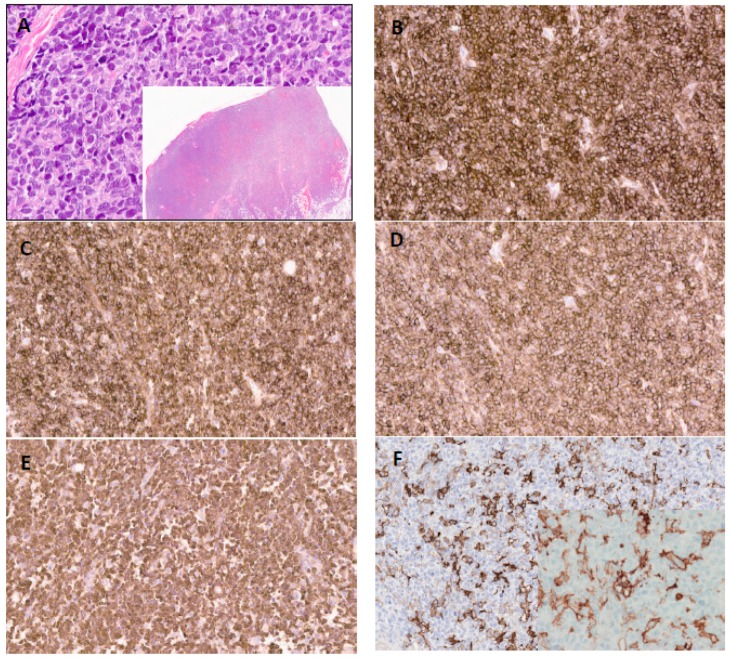
Excisional biopsy of cutaneous blastic plasmacytoid dendritic cell neoplasm lesion. Representative hematoxylin and eosin sections (high power ×200 and inset; low power ×30) (**A**) and immunohistochemical staining (×200) showing expression of CD4 (**B**), CD123 (**C**), CD56 (**D**), and TCL1 (**E**). Immunohistochemistry for and PD-L1 demonstrated partial to complete membranous staining with strong (3+) intensity (200x; inset 400×) in 55% of tumor cells (**F**).

**Figure 2:**
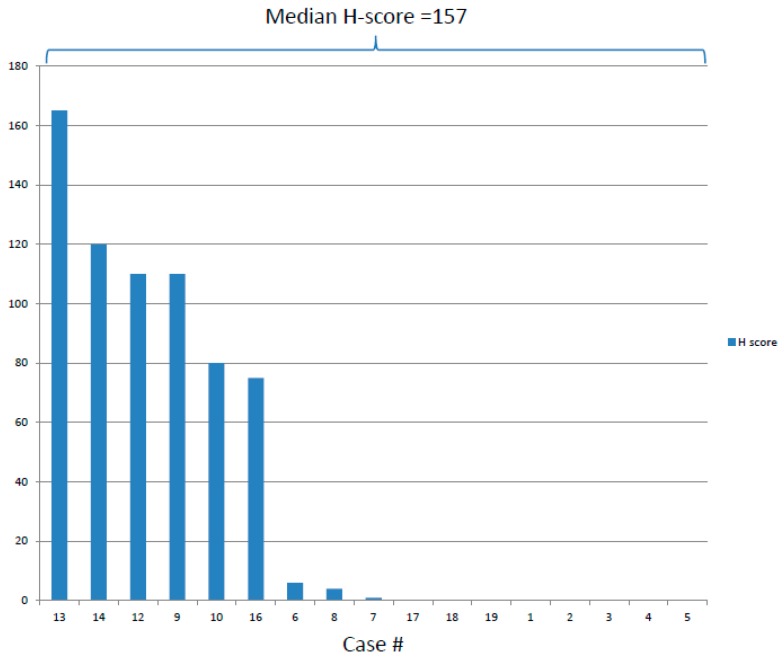
PD-L1 expression H-scores based on tumor cell staining by immunohistochemistry using the 22C3 antibody clone.

**Figure 3:**
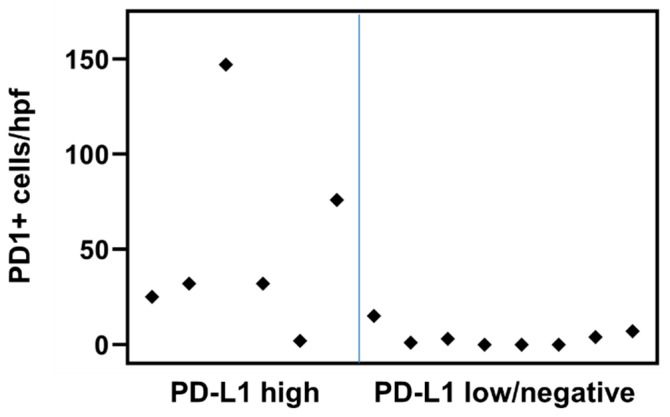
Comparison of the number of PD1-positive cells per high-power field between blastic plasmacytoid dendritic cell tumors with high-level PD-L1 expression (H-score ≥60) and those with negative/lowPD-L1 expression.

We tested 5 BPDCN cases for PD-L1 expression using both the 22C3 and 28-8 anti-PD-L1 monoclonal antibody clones, and they showed concordant staining. Since the former results in less background reactivity, the remaining patients were subsequently tested with the 22C3 clone. In all, PD-L1 protein expression status was determined on 28 patients, of whom 10 (47.6%) had positive PD-L1 staining on tumor cells. No PD-L1 expression by infiltrating, reactive lymphocytes was detected, although staining was observed in scattered macrophages in tissue samples and megakaryocytes in bone marrow samples. The extent of tumor cell staining ranged from 1% to 55%. Among the 10 cases with PD-L1 positivity, the median H-score was 157 (range, 1–165) (Figure 1 and Figure 2). The H-score was ≥60 in 7 cases and <10 in 3 cases. PD1 expression was evaluated in 20 patients and demonstrated a broad range of PD1-positive cells within BPDCN, with an overall average of 19.4 cells/hpf (median 4.5 cells/hpf; range, 0–147). We identified increased PD1-positive cells in most cases with high-level PD-L1 expression (*p* = 0.0211; *r* = 0.608). Namely, the 7 cases with an H-score ≥60 had a median of 32 PD1-positive cells/hpf (range, 2–147), whereas all other cases had a median of 2 cells/hpf (range, 0–15). (Figure 3)

Notably, longitudinal evaluation of PD1 and PD-L1 expression in one patient demonstrated commensurate increase in both within cutaneous lesions after treatment with tagraxofusp (SL-401). Namely, PD-L1 went from negative to high-level positive (>50% of tumor cells), while the number of PD1-positive cells went from 4.4/hpf to 25.2/hpf.

### 2.2. PD-L1 and PD1 Expression by RNAseq

**Figure 4:**
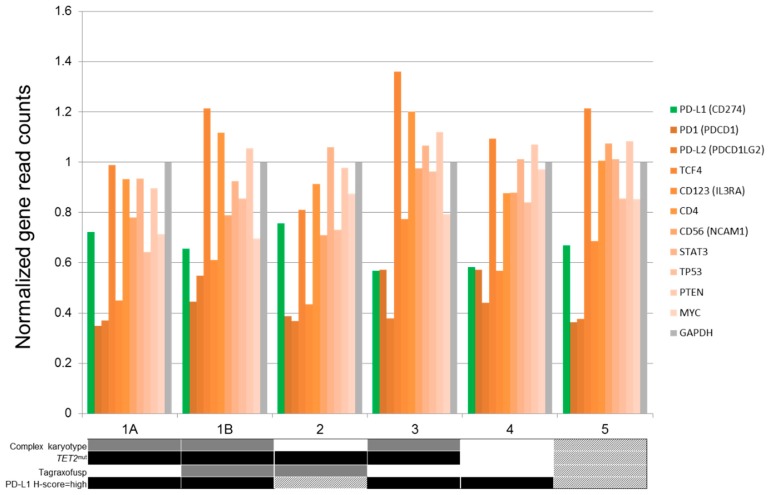
Normalized mRNA gene read counts (variance stabilizing transformation) relative to GAPDH of PD-L1 (CD274) and related transcripts of known significance in the PD1/PD-L1 axis. Samples 1A and 1B are from the same patient, collected at baseline (pretherapy) and at relapse following tagraxofusp therapy. Corresponding clinical and laboratory data are depicted schematically as follows: solid gray or black boxes = positive; while white boxes = negative; shaded boxes = unavailable.

We evaluated normalized gene read counts generated by RNAseq expression profiling data for expression of PD1 and PD-L1 in 6 BPDCN samples from 5 patients. In three samples with both expression and IHC data, PD-L1 mRNA levels correlated with expression in tumor cells by IHC. Samples collected from the same patient at baseline (pretherapy) and upon relapse following therapy with tagraxofusp demonstrated no significant interval changes in PD-L1 expression. The expression profile of genes biologically related to the PD-L1 axis in the samples analyzed by RNAseq is summarized in Figure 4.

### 2.3. Clinical and Molecular Correlates of PDL1 Expression

The study group characteristics are detailed in Table 1. Patients included 22 men (78.6%) and 6 women with a median age of 66.8 years (range: 23–87 years). Most patients were treated with hyper-CVAD (hyperfractionated cyclophosphamide, vincristine, doxorubicin, and dexamethasone alternating with high-dose methotrexate and cytarabine) (*n* = 11) or CHOP (cyclophosphamide, doxorubicin, vincristine, and prednisone) (*n* = 1) with or without stem cell transplant [25]. Fifteen patients in the study group were treated with tagraxofusp (anti-CD123) as part of a clinical trial (#NCT00397579) at our institution, followed by stem cell transplant in five of those patients. Two patients were treated with adjuvant radiotherapy and one patient passed away before any treatment. Four of our patients had concomitant or progression to other neoplasms, including acute myeloid leukemia (AML), chronic myelomonocytic leukemia, and myelodysplastic syndrome. Three of these patients showed PD-L1 expression with H-scores ranging from 60 to 110. One sample from the patient with disease progression to AML did not express PD-L1 protein.

The comparative characteristics of BPDCN patients with and without PD-L1 expression by IHC are summarized in Table 1. No correlation was identified between PD-L1 expression and age, gender, skin/lymph node/bone marrow involvement, peripheral blood parameters, cytogenetic features, mutation profile, number of PD1-positive cells, and death incidence. Although the median overall survival (OS) for patients with PD-L1 + BPDCN was lower than that of patients with PD-L1 disease, the difference was not significant (12 vs. 23 month; *p* = 0.743).

## 3. Discussion

In this study, we demonstrate PD-L1 expression by tumor cells in 47.6% of patients with BPDCN, suggesting a possible role for therapeutic inhibition of the PD1/PD-L1 axis in patients with this disease.

BPDCN is an aggressive disease with a poor prognosis similar to that of other acute leukemias, including AML and a subset of high-risk acute lymphoblastic leukemia (ALL). From the initial presentation with skin lesions, most patients with BPDCN will progress to a terminal leukemic phase within 12–24 months. In up to 20% of patients with BPDCN, AML, usually with myelomonocytic differentiation, may occur before the diagnosis of BPDCN or may develop at time of progression or relapse of BPDCN [26,27,28]. In this study, 3 (16%) patients developed AML, two of whom died of disease despite intensive treatment with chemotherapy and allo-SCT. Interestingly, all 3 patients showed PD-L1 H-scores >60.

Most treatment options for BPDCN employed to date have been extrapolated largely from regimens for other acute leukemias, such as ALL and AML [25,29]; these regimens include multi-agent intensive chemotherapy (e.g., cyclophosphamide, doxorubicin, vincristine, and prednisone as well as hyper-CVAD), central nervous system prophylaxis, radiation therapy, and stem cell transplant. Novel therapeutic modalities using antibody-based targeted approaches against BPDCN-specific markers such as anti-CD123 therapy are currently under investigation [30]. Exploration of additional therapeutic approaches, including the use of immune checkpoint inhibitors either as monotherapy or adjunct therapy to potentiate the effect of other classes of drugs seems warranted based on observations in this study.

Expression of PD-L1 in carcinomas derived from the stomach, liver, kidney, esophagus, pancreas, and bladder is associated with poor clinical outcomes. In contrast, PD-L1 expression correlates with better clinical outcomes in patients with breast cancers and Merkel cell carcinoma. The prognostic value of PD-L1 expression in lung and colorectal carcinomas and melanoma is controversial [20]. These findings demonstrate that the PD1/PD-L1 axis plays an important role in tumor immune evasion in many, but not all, tumor types. In this study, BPDCN patients with PD-L1 expression tended to have more favorable OS than those without PD-L1 expression, although the difference was not significant. However, we also found that BPDCN patients with high levels of PD-L1 protein expression (H-score ≥60) showed a trend toward worse OS and progression to AML compared those with low levels of PD-L1 (H-score < 60), warranting future validation of this finding in a larger patient cohort. Although it is acknowledged that the significance of PD-L1 expression in BPDCN as a biomarker for immune therapy selection remains to be determined, our data suggest that anti–PD-L1 therapy might be a consideration, particularly in BPDCN patients with higher levels of PD-L1 expression. It is worth noting that the relevance of PD-L1 expression on malignant plasmacytoid dendritic cells in BPDCN as a predictor of response to anti-PD1 therapy is still unknown. Furthermore, the role of PD-L1 expression in clinical outcome of patients with hematologic malignancies seems to be more complicated than in solid tumors. In classical Hodgkin lymphoma, PD-L1 is constitutively expressed, often as a result of genomic amplification, and it has one of the highest response rates to anti-PD1 therapy among hematologic malignancies. In multiple myeloma, on the other hand, although PD-L1 expression on plasma cells and dendritic cells has been reported [23], several anti-PD1 Phase 3 clinical trials have been put on hold by the United States Food and Drug Administration for lack of efficacy and excessive or unpredictable toxicity [31].

RNAseq (whole-transcriptome RNA sequencing) is widely used to analyze the status of cellular transcriptome. In this study, we analyzed a subset of samples to confirm PD-L1 protein expression analyzed by IHC and showed that PD-L1 IHC results correlated well with PD-L1 mRNA levels determined by whole-transcriptome mRNA sequencing. Furthermore, we present relative transcript levels of genes associated with the PD1/PD-L1 axis in other cellular systems.

## 4. Materials and Methods

### 4.1. Patient Group

The study group included 28 patients treated at The University of Texas MD Anderson Cancer Center (UTMDACC) between January 2000 and July 2018. All patients fulfilled the diagnostic criteria of BPDCN as defined in the World Health Organization Classification [32]. Inclusion in this study was based on the availability of archived tissue material, namely FFPE tissue blocks or unstained FFPE tissue sections adequate for PD1/PD-L1 immunohistochemistry (IHC), and/or frozen tissue adequate for RNA next-generation sequencing (RNAseq). Frozen tissue samples from 2 patients treated at The University of Texas at Southwestern were included for RNAseq studies only. With the exception of 2 samples obtained from patients who were treated prior to presentation to our institution, cases evaluated in this study represented untreated disease. For all patients, the diagnosis of BPDCN was confirmed as part of clinical workup by histologic evaluation and immunophenotypic analysis using IHC and/or multicolor flow cytometry (FC) analysis that included CD4, CD56, CD123, and TCL1 (IHC only), as described previously [33]. Relevant clinical and laboratory data were obtained by review of electronic medical records. The study was approved by the UTMDACC Institutional Review Board and conducted in accordance with the Declaration of Helsinki.

### 4.2. Evaluation of PD1 and PD-L1 Expression by Immunohistochemistry

PD-L1 protein expression was assessed using commercial United States Food and Drug Administration-approved companion diagnostic IHC assays in a clinical laboratory certified in accordance with the Clinical Laboratory Improvement Act [34]. PD-L1 IHC was performed using mouse (clone 22C3; Dako, Carpinteria, CA, USA) or rabbit (clone 28-8; Dako) monoclonal antibodies. PD1 IHC was performed using a mouse monoclonal antibody (clone MRQ-22; Cell Marque, Rocklin, CA, USA). Staining was performed on automated Leica Bond immunostainers (Leica Biosystems, Buffalo Grove, IL, USA) per manufacturer’s recommendations. PD-L1 and PD1 stains were interpreted independently by three pathologists (PPA, NS, JDK), and discordant results were resolved by consensus. PD-L1 expression was determined semi-quantitatively on the basis of viable tumor cells showing partial or complete membrane staining and categorized as positive if staining was detected in ≥1% tumor cells; tumor cells were detected and localized using CD123, CD56 and/or TCL1 stains. The H-score was calculated, defined as the intensity of positive staining (scale 1–3) multiplied by the percentage of positively staining tumor cells. The number of PD1-positive cells in BPDCN was determined quantitatively by averaging the number of positively staining cells in 5 randomly selected high-power fields (hpf) at 400× *g* on an Olympus BX41 microscope (Olympus, Tokyo, Japan).

### 4.3. Molecular Analysis

Mutation profiling was performed on a subset of cases (*n* = 14) using DNA extracted from bone marrow aspirate material using a next-generation (NGS) assay on the Illumina MiSeq sequencer (Illumina, San Diego, CA, USA) as described previously [35]. The assay detects mutations in hotspot genomic loci for the following genes: *EZH2*, *DNMT3A*, *GNAS*, *IDH1*, *IDH2*, *JAK2*, *KIT*, *KMT2A* (*MLL*), *MPL*, *NPM1*, *NOTCH1*, *NRAS*, *KRAS*, and *TP53*. The assay also assesses the exonic regions of the following genes: *ABL1*, *EGFR*, *GATA2*, *IKZF2*, *MDM2*, *NOTCH1*, *RUNX1*, *ASXL1*, *EZH2*, *HRAS*, *JAK2*, *KMT2A*, *NPM1*, *TET2*, *BRAF*, *IDH1*, *KIT*, *NRAS*, *TP53*, *DNMT3A*, *GATA1*, *IDH2*, *KRAS*, *MYD88*, *PTPN11*, and *WT1*.

### 4.4. Whole-Transcriptome RNA Sequencing

Whole-transcriptome RNA sequencing was performed on the SOLiD platform (ThermoFisher Scientific, Carlsbad, CA, USA) on a subset of cases (*n* = 4) using total RNA extracted from BM aspirate material to determine the levels of PD-L1 and PD1 mRNA transcripts. Briefly, 1 µg of total RNA from unsorted bone marrow (*n* = 3) or peripheral blood (*n* = 1) samples with >30% blasts was rRNA-depleted and purified using the Invitrogen RiboMinus™ eukaryotic kit (PN A1083708) and the components from RiboMinus Concentration module (Invitrogen, Carlsbad, CA, USA). Whole transcriptome RNA sequencing library construction was performed according to Life Technologies standard protocol (PN4452437 Rev. B) using SOLiD™ total RNAseq kit (PN4445374, Life Technologies, Carlsbad, CA, USA). Barcoded sequencing libraries were constructed, quantified, and pooled in equal molar ratios for sequencing template preparation performed using SOLiD™ EZ™ beads system. Whole transcriptome sequencing of mRNA was performed using SOLiD™ 5500XL system (Applied Biosystems, Foster City, CA, USA). The raw sequencing data was processed by LifeScope^TM^ Genomic Analysis Software (https://www.thermofisher.com) to produce the gene read counts. Then the R package DESeq was used to normalize the gene read counts. The variance stabilizing transformation (vsd) generated from DESeq were used for further analyses. The most variable genes in the heat map were selected by the Median Absolute Deviation of each gene. The R packages in Bioconductor (https://www.bioconductor.org/), a public available statistical computing tool for bioinformatics analysis, were utilized for data analysis.

### 4.5. Conventional Cytogenetics

Cytogenetic analysis was performed on diagnostic BM aspirate material using standard trypsin-Giemsa banding techniques as described previously [36]. Karyotypes were reported according to the 2016 International System for Human Cytogenetic Nomenclature [37].

### 4.6. Statistical Analysis

Fisher’s exact test and Mann-Whitney U test were used to assess categorical and continuous variables, respectively. The Pearson method was used to assess correlations between variables. The Kaplan-Meier method was used to estimate OS, and comparison of groups was performed using the Breslow (generalized Wilcoxon) test. Overall survival was calculated as the time from the date of BPDCN diagnosis to the date of last follow-up or death of any cause, whichever occurred first. Confidence intervals are provided to the 95th percentile (95% CI) where relevant. A *p*-value of <0.05 was considered statistically significant. Statistical analysis was performed using IBM SPSS Statistics 22.0 (IBM, Armonk, NY, USA).

## 5. Conclusions

In this study, we show that PD-L1 is variably expressed in BPDCN in a sizeable subset of patients. The therapeutic implication of this finding remains to be determined, but the results of this retrospective analysis provide a rationale for the exploration of immune checkpoint inhibitors in in BPDCN.

## Figures and Tables

**Table 1:** Study group characteristics.

Variable	Entire Group	PD-L1 Positive	PD-L1 Negative	*P*-Value
**N (%)**	28	10/21 (47.6)	11/21 (52.4)	
**Age in years, median (range)**	66.8 (22.8–86.7)	66.2 (28.7–87)	65.2 (38.5–84.7)	0.860
**Sex, N (%)**				0.635
Female	6 (21.4)	3	2	
Male	22 (78.6)	7	9	
**Anatomic location at diagnosis, N (%)**				0.643
Skin	23 (81.2)	9	9	
Bone marrow	19 (67.8)	5	9	
Lymph node	8 (28.6)	2	4	
**Peripheral blood parameters**				
White blood count (× 10^9^/L), median (range)	5.3 (1.7–76.5)	7.1 (2.0–76.5)	5.1 (2.4–13.3)	0.260
Hemoglobin concentration (g/dL), median(range)	12.2 (6.8–17.0)	12.1 (8.7–14.6)	13.1 (6.8–16.2)	0.622
Platelet count (× 10^9^/L), median(range)	147.5 (22–396)	178 (53–396)	183 (22–294)	0.888
**Bone marrow blast percentage, median (range)**	30 (4–100)	40 (14–70)	47 (5–95)	0.648
Cytogenetics, N (%) *				0.065
Diploid	8/19 (42.1%)	3/5	1/6	
Complex	7/19 (36.8%)	1/5	5/6	
**Mutations, present, N (%)**				1.000
*TET2*	7/17 (41.2)	4	2	
*ASXL1*	2 (11.8)	0	0	
*TET2 + ASXL1*	6 (35.3)	4	1	
*Other*	2 (11.8)	0	0	
**PD1 #/high-power field, median (range)**	4.5 (0–147)	14 (2–48)	18 (4–66)	0.123
**Tagraxofusp (SL-401) therapy, N (%)**	15/21 (71.4)	9/15 (60)	6/15 (40)	0.526
**Stem cell therapy, N (%)**	12/20 (60)	6/10 (60)	6/10 (60)	1.000
**Follow up duration in months, median (range)**	12 (2–66)	14 (2–48)	18 (4–66)	0.621
**Death, N (%)**	19 (67.9)	5/10 (50)	9/11 (81.8)	0.183

* Cases with bone marrow involvement. Complex defined as ≥3 structural abnormalities.
